# Disassortative Age-Mixing Does Not Explain Differences in HIV Prevalence between Young White and Black MSM: Findings from Four Studies

**DOI:** 10.1371/journal.pone.0129877

**Published:** 2015-06-19

**Authors:** Jeremy Alexander Grey, Richard B. Rothenberg, Patrick Sean Sullivan, Eli Samuel Rosenberg

**Affiliations:** 1 Department of Epidemiology, Rollins School of Public Health, Emory University, Atlanta, Georgia, United States of America; 2 Institute of Public Health, Georgia State University, Atlanta, Georgia, United States of America; The University of New South Wales, AUSTRALIA

## Abstract

**Objective:**

Age disassortativity is one hypothesis for HIV disparities between Black and White MSM. We examined differences in age mixing by race and the effect of partner age difference on the association between race and HIV status.

**Design:**

We used data from four studies of MSM. Participants reported information about recent sexual partners, including age, race, and sexual behavior. Two studies were online with a US sample and two focused on MSM in Atlanta.

**Methods:**

We computed concordance correlation coefficients (CCCs) by race across strata of partner type, participant HIV status, condom use, and number of partners. We used Wilcoxon rank-sum tests to compare Black and White MSM on partner age differences across five age groups. Finally, we used logistic regression models using race, age, and partner age difference to determine the odds ratio of HIV-positive serostatus.

**Results:**

Of 48 CCC comparisons, Black MSM were more age-disassortative than White MSM in only two. Furthermore, of 20 comparisons of median partner age, Black and White MSM differed in two age groups. One indicated larger age gaps among the Black MSM (18-19). Prevalent HIV infection was associated with race and age. Including partner age difference in the model resulted in a 2% change in the relative odds of infection among Black MSM.

**Conclusions:**

Partner age disassortativity and partner age differences do not differ by race. Partner age difference offers little predictive value in understanding prevalent HIV infection among Black and White MSM, including diagnosis of HIV-positive status among self-reported HIV-negative individuals.

## Introduction

Black men who have sex with men (MSM) represent a disproportionately high percentage of incident HIV infections among MSM in the United States,[1] and the disparity is greatest between young Black and White MSM.[2,3] Studies consistently show that HIV risk behaviors do not differ between White and Black MSM,[4–7] and several hypothesized explanations for the disparity in serostatus[4] remain inconclusive.[8] One hypothesis is that disassortative age mixing is more common among Black MSM in the United States.[4,8,9]

Assortativity, often termed “homophily,” is like-with-like pairing according to common characteristics. Disassortativity is mixing across strata or between groups. The type of mixing in sexual networks influences how disease transmission remains within or occurs between groups. In the context of HIV disparities, racial/ethnic assortativity may explain in part why incidence remains higher among Black MSM.[10] In addition, age disassortativity in young MSM would expose them to cohorts of MSM with greater HIV prevalence. If age mixing were more common among young Black MSM than among their White peers, it would place young Black MSM at a greater risk of infection. Thus, age disassortativity could explain why young Black MSM have greater HIV incidence than White MSM in the same age range.[11–13]

Although partnering with older men increases the risk of HIV exposure to young MSM, it is not firmly established that age-disassortative partnerships are more common among young Black MSM compared to young White MSM. Furthermore, even if young Black MSM engage in age-disassortative partnerships more frequently than young White MSM, it is also unknown whether differences in age mixing play a role in HIV disparities between the subpopulations. In this analysis, we use data from four studies of MSM to examine the extent to which Black and White MSM partnerships have different age-mixing patterns. We seek to identify whether partner age differences account for some portion of the difference in HIV prevalence between White and Black young MSM.

## Methods

### Studies

Our data are from four studies of MSM conducted at Emory University from 2009–2013. Two studies were Internet-based samples in the US. Two others sampled MSM in the Atlanta area, of which one used a mixture of online and offline recruitment methods and the other used offline methods exclusively. For all studies, participants were eligible if they were male at birth, aged 18 years or older, and had a male sex partner within a specified period prior to enrollment in the study. In order to examine differences in age mixing between Black and White MSM, we restricted our analytic samples to those who reported being Black or White and not Hispanic. All studies were reviewed and approved by the Emory University Institutional Review Board.

The first online study, Barriers to Online Prevention Research (BOPR), was a cross-sectional Internet-based study conducted in 2009.[14] A total of 9,005 surveys were collected using banner advertisements placed on MySpace.com, a social network site. The second online study, Checking In, conducted in 2010, used a similar recruitment strategy that included social network sites and one dating Web site.[15] Individuals eligible for the baseline questionnaire were male, at least 18 years old, and had a male sex partner in the past 12 months. Following the administration of an online consent document, participants completed a 60-minute baseline questionnaire.

InvolveMENt is a prospective cohort study of racial disparities in HIV prevalence and incidence conducted in Atlanta, Georgia from 2010–2014.[16,17] It measured factors possibly related to HIV prevalence on individual, dyadic, and community levels. Recruitment was designed to yield a balanced sample of Black and White MSM. Subjects were recruited primarily using venue-time-space sampling, with some men recruited via banner ads posted on Facebook. Venues used for recruitment were bars, clubs, gyms, restaurants, special events, and bathhouses. Staff approached men at these venues and invited them to participate. Facebook ads were broadcast to men over the age of 18 who lived in the Atlanta area and expressed an interest in men on their profile. After three months, we restricted enrollment to men under the age of 40.[17] For this analysis, we used data from the baseline survey only.

Finally, the MAN Project was a cross-sectional study of the sexual networks of Black and White MSM in the Atlanta metropolitan area.[18] Seed respondents were recruited at venues frequented by MSM, using venue-time-space sampling,[19] and these seed participants referred sex partners for enrollment in the study. Seeds were eligible if they were Black or White, not Hispanic, under the age of 40, had a male sex partner in the previous 3 months, and provided two methods of contact. Eligibility for referred partners was similar, but was not restricted by age or race/ethnicity. For analysis, we used only seeds and enrolled partners who were Black or White and non-Hispanic.

### Measures

All studies used similar computer-assisted survey instruments, administered using SurveyGizmo v2.6.[20] All participants reported their age, race, ethnicity, and HIV status and answered questions about their recent male sexual partners using a partner-by-partner inventory. We asked about the most recent partner in the past 12 months in BOPR; the most recent 5 partners in the past 6 months in Checking In and InvolveMENt; and the most recent 10 partners in the past 12 months in the MAN Project. Partner characteristics included age, race, and HIV status (HIV-positive,-negative, or-unknown) based on the respondent’s report. For each partner, respondents also reported whether the partner was a main partner or a casual partner, whether they engaged in anal intercourse the last time they had sex, and, if so, whether a condom was used.

Individuals who were uncertain of their partner’s exact age were asked to estimate it relative to their own age, according to the following options: “more than 10 years younger,” “two to 10 years younger,” “within one year,” “two to 10 years older,” and “10 or more years older.” To generate conservative partnership age-gap estimates, we used the bound closest to the participant’s age to estimate partner age, e.g., “two to 10 years younger” was recoded as the partner’s age minus two years. Most participants (96.8%) reported exact ages, and the proportion estimated did not differ by race (*χ* ^*2*^ = 0.447, *p* = 0.50).

Self-reported HIV status was collected in all studies and was used as a dependent variable in primary analyses. Participants in InvolveMENt and MAN Project also received HIV testing. Consequently, for these two projects, we conducted sub-analyses using laboratory-diagnosed HIV status.

### Statistical Methods

Because of the different designs and populations (e.g., US and Atlanta MSM), we analyzed differences by study. We used Lin’s concordance correlation coefficient (CCC) as a global measure of age assortativity across dyads.[21–23] For Black and White MSM within each study, we computed CCCs and their standard errors, using a SAS macro.[24] We further stratified CCCs by one of several variables: partner type (main or casual), condom use at last sex (yes or no), and self-reported HIV status (negative, positive, and unknown). To examine the potential influence of repeated partnership observations from participants, we also examined CCCs among those who reported one, two, three, four, or five or more partners. We used *t*-tests to evaluate significant differences between Black and White men in each stratum. Given the number of comparisons and the increased risk of Type I errors, statistical significance was evaluated at α = 0.01. To more finely understand the distribution of within-partnership age differences, we also computed median age differences between participants and their male partners in three age categories: 18–19, 20–24, and 25–29.[25,26] We further stratified these comparisons by self-reported HIV status. We then used Wilcoxon rank-sum tests to determine significant differences by race in each stratum.

Finally, to understand the relationship between partner age and HIV prevalence among young MSM (i.e., under the age of 30) and the degree to which partner age might account for racial disparities in prevalence, we conducted a series of logistic regressions estimating HIV status, both self-reported and laboratory-diagnosed, among MSM aged 18 to 29. In a third scenario, we also restricted the latter analysis to individuals who self-reported being HIV-negative upon enrollment, prior to being tested during the study. All models included a random effect for study to account for differences in study design and population, as well as fixed effects for participant age, participant race, and age difference between participant and his partner. To compare our findings to previous studies,[27–29] age difference was considered three ways: as a continuous variable, as “five or more years older” (dichotomous), and as “ten or more years older” (dichotomous). Because the objective was to understand risk of HIV acquisition by younger men from older men, all of these were signed.

For each partner age difference definition and outcome (self-reported HIV status, laboratory-diagnosed HIV status, and laboratory-diagnosed HIV status among self-reported HIV-negative individuals), we fit four models to examine the influence of control for partner age on the estimated relative odds of HIV infection by race. In Model 1, we included only participant age and race. In Model 2, we added partner age difference. In Model 3, we included additional potential confounders of the participant race-HIV relationship: partner race, partner HIV status, and whether they engaged in unprotected anal intercourse (UAI) at last sex. Finally, Model 4 was the same as Model 3 but excluded partner age difference. We considered a >10% change in odds ratio of HIV infection by race to be indicative of meaningful confounding.[30] We conducted all analyses using SAS 9.3.

## Results

We obtained participant and partner age information for 13,634 dyads from 7,393 non-Hispanic Black and White participants across the four studies. Of these dyads, 9,284 (74.6%) were from White participants and 4,350 (25.4%) were from Black participants (Table 1).

**Table 1:** Number of non-Hispanic Black and White participants (N = 7,393) and number of partners reported (N = 13,634) in four studies of men who have sex with men in the United States, by study, participant age group and participant race/ethnicity.

		*BOPR*		*Checking In*		*InvolveMENt*		*MAN Project*	
		Enrolled n (%)	Partners n (%)	Enrolled n (%)	Partners n (%)	Enrolled n (%)	Partners n (%)	Enrolled n (%)	Partners n (%)
**TOTAL**		3980 (54%)	3980 (29%)	2335 (32%)	5858 (43%)	785 (11%)	2601 (19%)	293 (4%)	1195 (9%)
**AGE**	**RACE**								
**18–19**	**White**	858 (22%)	858 (22%)	165 (7%)	394 (7%)	16 (2%)	45 (2%)	1 (0%)	3 (0%)
	**Black**	269 (7%)	269 (7%)	62 (3%)	160 (3%)	27 (3%)	88 (3%)	10 (3%)	28 (2%)
**20–24**	**White**	1023 (26%)	1023 (26%)	445 (19%)	1065 (18%)	90 (11%)	316 (12%)	33 (11%)	186 (16%)
	**Black**	396 (10%)	396 (10%)	190 (8%)	446 (8%)	154 (20%)	500 (19%)	69 (24%)	270 (23%)
**25–29**	**White**	468 (12%)	468 (12%)	322 (14%)	775 (13%)	105 (13%)	389 (15%)	30 (10%)	137 (11%)
	**Black**	172 (4%)	172 (4%)	91 (4%)	231 (4%)	130 (17%)	410 (16%)	47 (16%)	139 (12%)
**30–39**	**White**	368 (9%)	368 (9%)	372 (16%)	985 (17%)	120 (15%)	422 (16%)	42 (14%)	214 (18%)
	**Black**	117 (3%)	117 (3%)	101 (4%)	254 (4%)	119 (15%)	346 (13%)	49 (17%)	180 (15%)
**40+**	**White**	253 (6%)	253 (6%)	479 (21%)	1300 (22%)	15 (2%)	54 (2%)	7 (2%)	29 (2%)
	**Black**	56 (1%)	56 (1%)	108 (5%)	248 (4%)	9 (1%)	31 (1%)	5 (2%)	9 (1%)

**Note**: Percentages for the *total* row are row percentages across studies (e.g., percentage of enrolled participants from the *BOPR* study out of all enrolled participants). The remaining percentages in the table are column percentages within study (e.g., percentage of partners reported by Black MSM aged 18–19 out of all partners reported in a given study).

**Fig 1:**
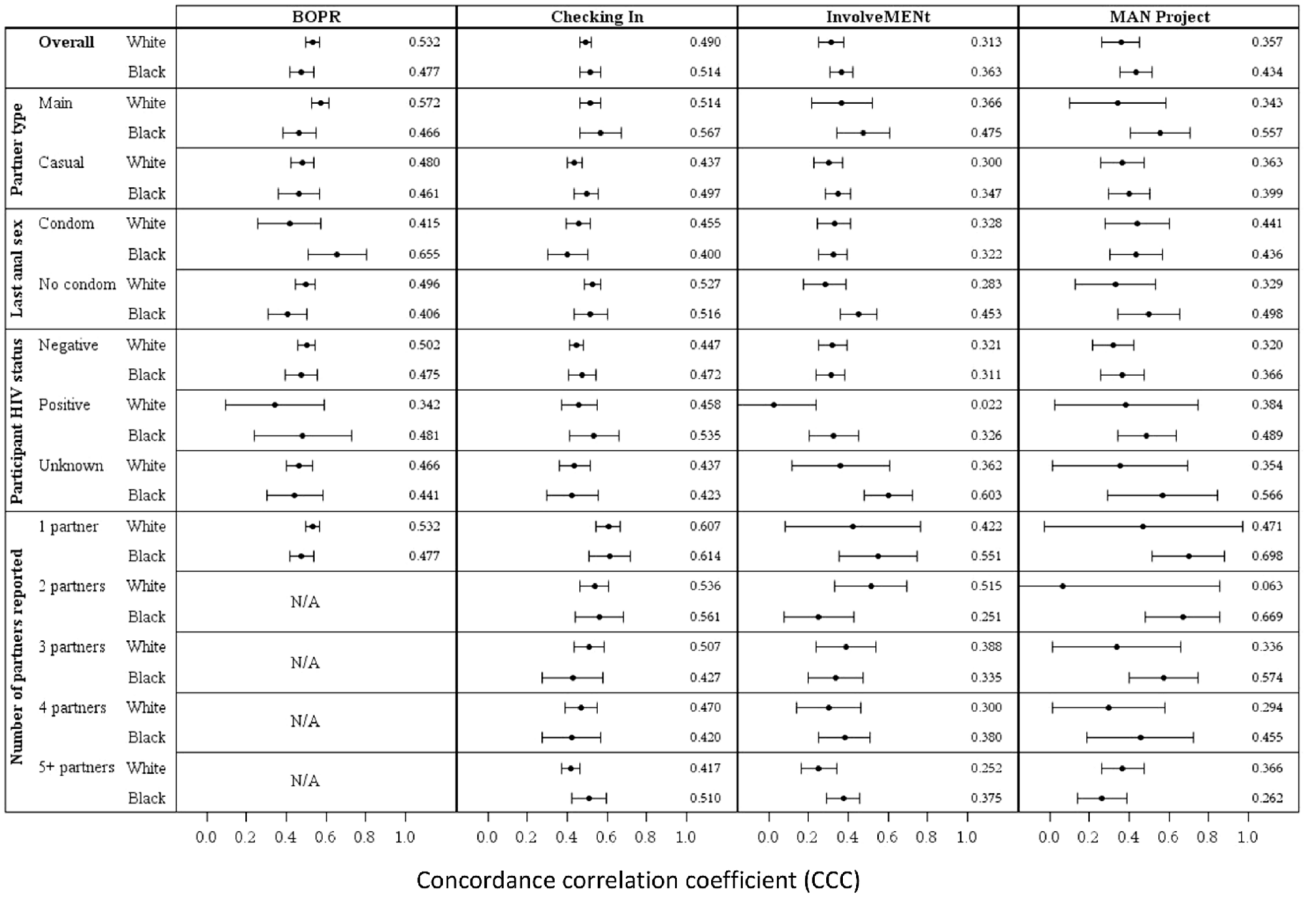
Concordance correlation coefficients for participant and partner age, by study, race and select characteristics. Note: CCCs are depicted with 99% confidence intervals. Point estimates are listed on the right side of each cell. Although values range from -1 to 1, only the range from 0 to 1 is depicted.

CCCs were almost all positive, indicating that MSM were age-assortative with their sexual partners, although the extent of assortativity varied by stratum. The 99% confidence intervals included zero for HIV-positive White MSM in the InvolveMENt study and White MSM with two partners in the MAN Project (Fig 1). Confidence intervals for several categories were wide, probably because of small sample sizes. For the 48 CCC comparisons examined, few were significantly different between Black and White MSM. Of the five that were significant at α = 0.01, three indicated greater age disassortativity among White MSM compared to Black MSM. A Wilcoxon signed-rank test indicated that significantly more of the 48 CCCs were lower for White MSM (p = 0.004), suggesting that White MSM are more age-disassortative than Black MSM overall.

We found no statistically significant overall difference in age mixing by race in any of the four studies. In BOPR, Black MSM were more age-disassortative with their main partners than White MSM (*p* = 0.004), while White MSM were more age-disassortative with partners with whom they used condoms. When examined by HIV status, age disassortativity was the same among HIV-negative Black and White men across all studies. In the InvolveMENt study, White men who were HIV-positive or did not know their HIV status were more age-disassortative than Black men of the same status (p = 0.002). White MSM in InvolveMENt were also more age-disassortative with their UAI partners than Black MSM (p = 0.002).

We observed few differences when we stratified the CCCs by race and number of reported partners, and only one was statistically significant: in the InvolveMENt study, Black men who reported two partners were more age-disassortative than White men, (p = 0.007). Within each partner number category, however, the pattern was similar for Black and White MSM (e.g., those with more partners are more age-disassortative, regardless of race).

We also computed CCCs for Black men with White partners and Black men with Black partners (data not shown). Black men were more age-disassortative with their White partners than with their Black partners in all studies, although none of the differences was statistically significant. In BOPR and Checking In, CCCs for White partners of Black men were 0.364 and 0.476, respectively, and CCCs for Black partners were 0.505 and 0.514. Age agreements for InvolveMENt and MAN Project were lower but were in the same direction, with CCCs of 0.287 and 0.337 for White partners and 0.371 and 0.487 for Black partners.

Men in the two youngest age categories (18–19 and 20–24 years) had older partners by 1 to 3.5 years on average, while those in the 25–29 age range had partners approximately their own age. Across studies and age strata, there were few significant differences in the median age of partners of Black and White men (Table 2).

**Table 2:** Median partner age difference by self-reported HIV status, race, and age group (N = 6,352).

		*BOPR*			*Checking In*			*InvolveMENt*			*MAN Project*		
Age	Race	n	Mdn (IQR)	p	n	Mdn (IQR)	p	n	Mdn (IQR)	p	n	Mdn (IQR)	p
**FULL SAMPLE**													
**18–19**	**White**	406	1 (0, 4)	0.815	190	2 (0, 6)	0.128	29	6 (2, 10)	0.327			
	**Black**	148	1.5 (0, 4)		76	3 (1, 9.5)		77	4 (1, 9)				
**20–24**	**White**	646	2 (-1, 6)	0.844	745	2 (0, 6)	0.618	287	3 (0, 9)	0.285	157	2 (0, 8)	0.099
	**Black**	263	2 (-1, 4)		306	2 (-1, 7)		419	3 (0, 8)		244	2 (0, 6)	
**25–29**	**White**	348	-1 (-4, 4)	0.131	656	0 (-4, 5)	0.704	377	2 (-2, 9)	0.051	134	1 (-3, 3)	0.645
	**Black**	129	0 (-3, 6)		214	1 (-3, 4)		372	1 (-2, 7)		129	0 (-3, 3)	
**HIV-NEGATIVE**													
**18–19**	**White**	404	1 (0, 4)	0.980	182	2 (0, 5)	0.080	27	5 (1, 11)	0.373			
	**Black**	145	1 (0, 4)		75	3 (1, 10)		72	4 (0.5, 8.5)				
**20–24**	**White**	634	1 (-1, 5)	0.955	712	2 (-0.5, 6)	0.604	264	3 (0, 8)	0.755	147	3 (0, 8)	0.067
	**Black**	242	1 (-1, 4)		275	2 (-1, 6)		352	3 (0, 8)		173	2 (0, 6)	
**25–29**	**White**	332	-1 (-4, 3)	0.178	622	0 (-4, 5)	0.486	333	2 (-2, 9)	0.027	124	0 (-3, 3)	0.515
	**Black**	114	0 (-3, 6)		159	1 (-3, 3)		257	1 (-3, 7)		90	0.5 (-2, 3)	
**HIV-POSITIVE**													
**18–19**	**White**	2	-0.5 (-1, 0)	0.222	8	5 (2, 12)	0.359	2	9 (8, 10)	1.000			
	**Black**	3	5 (2, 11)		1	0 (0, 0)		5	8 (6, 12)				
**20–24**	**White**	12	5.5 (1.5, 11)	0.578	33	3 (1, 9)	0.486	23	10 (6, 17)	0.002	10	-3 (-5, 4)	0.055
	**Black**	21	4 (0, 8)		31	2 (0, 8)		67	3 (1, 8)		71	2 (-1, 6)	
**25–29**	**White**	16	-1 (-3, 17)	1.000	34	0 (-2, 6)	0.929	44	1 (-3, 12.5)	0.931	10	4 (1, 8)	0.003
	**Black**	15	0 (-2, 11)		55	1 (-3, 6)		115	2 (-1, 7)		39	-1 (-4, 2)	

**Note**: Negative values indicate younger partner age.

For those ages 18–29—who contributed nearly two thirds of reported dyads (n = 8,768, 64.3%)—the odds ratios of being HIV positive among Black MSM were 3.2 (self-reported) and 5.0 (laboratory diagnosis) compared to White MSM (Table 3, Bivariate). Among individuals who self-reported being HIV-negative, the odds ratio of laboratory-diagnosed HIV-positive serostatus was 7.5 for Black MSM, compared to White MSM (Table 3, Bivariate). Adjusting for participant age increased the estimated odds of self-reported HIV-positive serostatus by 14.6% (Table 3, Model 1), because the White sample was slightly older. A similar increase occurred (15.3%) when we examined laboratory-diagnosed HIV status in the two Atlanta-based studies (Table 3, Model 1), although we did not observe a similar increase when we restricted the analysis to individuals who self-reported HIV-negative serostatus (0.2%; Table 3, Model 1).

**Table 3:** Crude and adjusted odds of HIV-positive serostatus, measured by self-report (4 studies, N = 6,262), laboratory diagnosis (2 studies, N = 2,438), and laboratory diagnosis restricted to self-reported HIV-negative MSM under the age of 30 (2 studies, N = 1,813).

	Bivariate		Model 1:Participant race + participant age		Model 2:Model 1 + partner age difference		Model 3:Model 2 + covariates†		Model 4:Model 3-partner age difference	
	OR	99% CI	aOR	99% CI	aOR	99% CI	aOR	99% CI	aOR	99% CI
**SELF-REPORTED STATUS** (N = 6,262)										
**Participant Black race** (ref: White)	3.21	2.51, 4.11	3.68	2.86, 4.73	3.74	2.91, 4.82	2.56	1.80, 3.65	2.57	1.81, 3.67
**Participant age**	1.16	1.12, 1.21	1.20	1.15, 1.24	1.21	1.16, 1.26	1.19	1.14, 1.24	1.18	1.13, 1.24
**Partner age difference**	1.01	0.99, 1.02			1.02	1.01, 1.04	1.01	0.99, 1.03		
**LABORATORY-DIAGNOSED STATUS** (N = 2,438)										
**Participant Black race** (ref: White)	4.97	3.70, 6.67	5.73	4.23, 7.77	5.83	4.30, 7.92	3.67	2.34, 5.72	3.67	2.35, 5.72
**Participant age**	1.09	1.04, 1.13	1.14	1.09, 1.19	1.15	1.10, 1.20	1.13	1.08, 1.19	1.13	1.13, 1.18
**Partner age difference**	1.00	0.98, 1.02			1.01	0.99, 1.03	1.01	0.99, 1.03		
**LABORATORY-DIAGNOSED STATUS** (self-reported HIV-negative MSM under 30, N = 1,813)										
**Participant Black race** (ref: White)	7.45	4.01, 13.8	7.47	4.00, 14.0	7.54	4.02, 14.1	2.61	1.17, 5.82	2.60	1.17, 5.80
**Participant age**	0.95	0.88, 1.02	1.00	0.93, 1.08	1.01	0.93, 1.09	1.01	0.93, 1.09	1.00	0.93, 1.09
**Partner age difference**	1.00	0.98, 1.02			1.01	0.97, 1.04	1.02	0.99, 1.05		

**Note**: All estimates are adjusted for study.

^†^Covariates include partner’s race, partner’s HIV status, and unprotected anal intercourse (UAI) at last sex.

Associations between partner age difference and HIV status were not significant for either self-reported or laboratory-diagnosed status, including laboratory-diagnosed status restricted to individuals who self-reported being HIV-negative. Although partner age difference was significantly associated with self-reported HIV status, after adjusting for participant race and age (Table 3, Model 2), no index of partner age difference was significant after adjusting for additional covariates (Table 3, Model 3).

When we modeled predictors of self-reported HIV-positive serostatus, including partner age difference in the model negligibly changed the race aOR (from 3.68 to 3.75, +1.9% change; Table 3, Model 2). The result was similar when we included partner age difference as a binary variable of either *5 or more years older* or *10 or more years older*, both of which resulted in a 1.4% increase (not shown). However, adjusting for partner race, partner HIV status, and UAI at last sex reduced the aOR by 31.4%, 31.7%, and 65.4% for self-reported status, laboratory-diagnosed status, and laboratory-diagnosed status among self-reported HIV-negative individuals, respectively (Table 3, Model 3), indicating that the race association was confounded by one or more of these variables. Subsequently removing partner age did not change these adjusted estimates greatly (<1% for each scenario; Table 3, Model 4).

## Discussion

HIV is more prevalent among older MSM.[17] Consequently, the nominal risk of HIV infection is greater with older sexual partners.[27,31–33] Unprotected anal intercourse (UAI) may also be more common among those with older partners,[29,34] which would exacerbate this effect. However, in four studies and over 13,000 partnerships, we found few differences in age mixing between Black and White MSM. Furthermore, partner age difference did not influence the odds ratio for Black MSM and HIV-positive serostatus among those under the age of 30, suggesting that partner race is not a major driver of HIV disparities in young MSM.

Our findings were consistent across the four studies despite differing geographic contexts of the study samples (US and Atlanta) and recruitment methods (Internet- and venue-based). There were several differences between the national and Atlanta studies, however, that may indicate that partnership formation patterns differ in the local context. Among the 68 comparisons of Black and White MSM—48 CCC and 20 median age comparisons—2 suggested that Black MSM are more age-disassortative than White MSM and 1 suggested that young Black MSM, aged 18–19, have older partners than their White peers. First, in the *BOPR* study, Black MSM were more age-disassortative with their main partners than White MSM. Given that an important proportion of HIV transmissions occur between main partners,[35] this finding might imply that age mixing occurs more often among Black MSM in circumstances that are potentially higher risk (e.g., more frequent unprotected anal intercourse). We did not find this difference in the other three studies, however, including the Atlanta-based studies, where racial disparities in HIV incidence among MSM are particularly high.[36]

In *Checking In*, the fact that Black MSM aged 18 to 19 had significantly older partners than White MSM in the same age group could mean that age disassortativity in the youngest Black MSM contributes to early HIV disparities. This comparison is only one of 20 stratified partner age comparisons across the four studies, the rest of which indicated no difference by race (α = 0.01). Our finding that age mixing patterns do not differ much between Black and White men contradict those of a study that found significant differences[28] but were consistent with one that found none.[7] Although it has been reported that Black MSM are more likely to have a partner 10 or more years older than White MSM,[28] this estimate was not adjusted for index age. A study that examined less extreme age mixing (2 or more years older) by age strata (18 to 24, 25 to 29, and 30 and older) found no differences between Black and White MSM.[7] In addition, differences from Berry et al.[28] may be due to the geographic context of their study; while our studies were either national or limited to Atlanta, theirs was based on data from San Francisco.

Few studies have directly examined the role of age disassortativity HIV disparities among Black and White MSM. Bingham et al.[37] found that adjusting for both partner age difference (“most partners 5 or more years older”) and partner race reduced relative odds of being HIV-positive (Black vs. White) by 20%, from 6.9 to 5.5.[37] However, it is unclear whether this change was due mostly to partner race. As a follow-up to Bingham et al.,[37] we included race and age difference of sexual partner, measured three ways, in separate steps. Doing this resulted in a minimal change in estimates of the association between race and HIV serostatus, either self-reported or laboratory-diagnosed, when partner age difference was added to the model. The findings were similar when we restricted our analysis to predict laboratory-diagnosed HIV-positive status among young MSM who reported being HIV-negative. Thus, we found no evidence that age difference confounds the association between race and HIV status. This result also did not differ whether we considered it as a continuous or dichotomous variable. It is possible that the finding initially reported in Bingham et al.’s[37] was due mostly to the effects of partner race and not difference in partner age.

Our study had several limitations. First, we modeled odds of prevalent HIV status, not of new HIV infection. As a result, we cannot determine whether having sex with older MSM occurred before, after, or as a result of HIV acquisition. Our cross sectional data suggest, however, that mixing patterns do not change substantially over the decade 20–29, and prevalent infection may be a reasonable surrogate. We saw no racial differences in age mixing among HIV-negative MSM. Second, we did not predict the odds ratios of HIV risk behavior such as UAI, as Newcomb and Mustanski[38] did, but included it in our model instead. Using HIV risk behavior as an outcome, Newcomb and Mustanski[38] found a three-way interaction that indicated higher odds of receptive UAI among young Black MSM who had older partners. However, meta-analyses suggest few behavioral differences between Black and White MSM.[4–5] Therefore, although risk behaviors lead to HIV transmission, there is little evidence to suggest that differences in HIV risk behavior drive racial disparities in HIV prevalence and incidence. Consequently, HIV status is a more appropriate outcome for the current analysis.

Another limitation was that we stratified our correlation coefficients in order to examine clustering among individuals who reported greater numbers of partners, but we did not directly account for correlation among the partners of the same participants. Earlier studies of age mixing in young MSM used proportion of partners who were older as a way around this, such as by categorizing participants as having “no older partners,” “some older partners,” and “exclusively older partners.”[12] Our analyses would not directly capture whether there was something unique about individuals who have exclusively older partners or whether there is a dose-response in terms of the proportion of partners who are older. Such analyses are difficult, however, because individuals would have to estimate the proportion of partners who were older. In our study, we used partner age for recent sexual partners and modeled the dyads rather than the individuals. In this way, age-differentials in dyads should contribute in proportion to their frequency in the data, such that those with a greater number of older partners who are HIV positive would tip the effect toward older partners as a risk factor.

## Conclusions

Despite the common hypothesis that age disassortativity is driving HIV disparities among MSM, data from four studies suggest that partner age does not affect estimates of racial HIV, including HIV prevalence among individuals who self-reported being HIV-positive. Our findings were true of samples collected in Atlanta, specifically, which has profound racial HIV disparities.[39] Given the large race differentials in HIV prevalence among young MSM, and the very high prevalence among young Black MSM, race assortativity may be more meaningful for HIV disparities than age disassortativity.[3,16] Young Black MSM who have sex with other young Black MSM are at higher risk than young White MSM who have sex with young White MSM. The amount of additional risk involved in having older partners may be minimal in comparison.
